# Pediatric orbital wall fractures: Prognostic factors of diplopia and ocular motility limitation

**DOI:** 10.1371/journal.pone.0184945

**Published:** 2017-11-02

**Authors:** Yung Ju Yoo, Hee Kyung Yang, Namju Kim, Jeong-Min Hwang

**Affiliations:** 1 Department of Ophthalmology, Kangwon National University Hospital, Kangwon National University Graduate School of Medicine, Chuncheon, Korea; 2 Department of Ophthalmology, Seoul National University College of Medicine, Seoul National University Bundang Hospital, Seongnam, Korea; Bascom Palmer Eye Institute, UNITED STATES

## Abstract

**Objectives:**

To investigate the factors affecting recovery of diplopia and limited ocular motility in pediatric patients who underwent surgery for orbital wall fracture.

**Design:**

Retrospective observational case series.

**Methods:**

In this retrospective observational case series, 150 pediatric patients (1–18 years old) who were diagnosed with orbital medial wall or floor fracture and underwent corrective surgery between 2004 and 2016 at Seoul National University Bundang Hospital were included. The medical records of patients with orbital medial wall or floor fracture were reviewed, including sex, age, diplopia, ocular motility, preoperative computed tomographic finding, and surgical outcomes. Factors affecting recovery of diplopia and ocular motility limitation were analyzed.

**Results:**

Of the 150 patients (134 boys; mean age, 14.4 years) who underwent corrective surgery for orbital wall fracture, preoperative binocular diplopia was found in 76 (50.7%) patients and limited ocular motility in 81 (54.0%). Presence of muscle incarceration or severe supraduction limitation delayed the recovery of diplopia. In case of ocular motility limitation, presence of muscle incarceration and retrobulbar hemorrhage were related with the delayed resolution. Multivariate analysis revealed supraduction limitation (Hazard ratio [HR] = 1.74, 95% confidence interval [CI] = 1.19–2.55), larger horizontal orbital floor defects (HR = 1.22, 95% CI = 1.07–1.38), and shorter time interval to first visit (HR = 0.73) as negative prognostic factors for the recovery of diplopia. In addition, muscle incarceration (HR = 3.53, 95% CI = 1.54–8.07) and retrobulbar hemorrhage (HR = 3.77, 95% CI = 1.45–9.82) were found as negative prognostic factors for the recovery of motility limitation.

**Conclusions:**

Presence of muscle incarceration and retrobulbar hemorrhage, horizontal length of floor fracture, supraduction limitation, and time interval from trauma to first visit were correlated with the surgical outcomes in pediatric orbital wall fracture patients. These results strengthen that the soft tissue damage associated with bony fracture affects the orbital functional unit. When managing children with orbital wall fracture, meticulous physical examination and thorough preoperative computed tomography based evaluation will help physicians to identify damage of orbital functional unit.

## Introduction

Orbital wall fractures are common results of periorbital blunt trauma [1]. In the pediatric population, the common presenting symptoms are quite different from adults because fractures with tissue incarceration are more common due to their bony elasticity [2–4]. Incarcerated orbital tissues frequently cause limited ocular motility and subsequent diplopia [5, 6], and in pediatric patients, these symptoms can be persistent even after relevant surgery [6, 7]. There had been studies reporting factors affecting surgical outcomes in adult orbital wall fracture patients [8]. Yu et al.[8] reported higher number of fractures and delayed surgical timing as poor prognostic factors of orbital wall fracture. However, to the best of our knowledge, other than a few small case series [6, 9–11], the prognostic factors of surgical outcome in pediatric orbital wall fractures has not been evaluated in a large scale. Previous study on pediatric wall fracture reviewed 87 cased of surgery, but only an increasing incidence of surgery in older patients were reported [12]. In the present study, we investigated diplopia and limitation of ocular motility in pediatric orbital wall fracture and analyzed the factors associated with surgical outcomes of pediatric orbital wall fracture.

## Materials and methods

This study adhered to the Declaration of Helsinki and the protocol was approved by the Institutional Review Board of Seoul National University Bundang Hospital (IRB No.: SNUBH B-1606-349-109). All clinical investigation was conducted according to the principles expressed in the Declaration of Helsinki. Informed consent was not given, as patient records and information were anonymized and de-identified prior to analysis. This study retrospectively reviewed the medical records of 150 pediatric patients (less than 18 years of age) who had received a diagnosis of orbital floor or medial wall fracture based on computed tomography (CT), and underwent corrective surgery between January 1, 2004, and April 30, 2016, at Seoul National University Bundang Hospital. Surgery was performed by 1 ophthalmologist, 1 otolaryngologist and 8 plastic surgeons. Patients who have pre-existing strabismus or limitation of ocular motility were excluded. In addition, patients were excluded if they were lost to follow up before 2 weeks postoperative.

Using electronic medical records and CT, we collected data on sex, age, diagnosis, symptoms and signs at first visit, cause of injury, time interval between injury and first visit, time interval between first visit and surgery, presence and extent of diplopia and ocular motility limitation, enophthalmos, fracture location, fracture type, fracture size, presence of tissue incarceration, presence of fat or muscle herniation, presence of retrobulbar hemorrhage and the timing of recovery of ocular motility limitation and diplopia. Limitation of ocular motility was graded on a numerical scale of 0 to -4, with 0 representing no limitation and -4 representing no movement in the field of gaze [13]. Patient-reported binocular diplopia was considered present when double vision was reported in any direction of gaze and was eliminated when the occluder is placed in front of either eye We also measured the field of binocular single vision with the Goldmann perimeter (Haag-Streit AG, Keoniz, Switzerland) using the III4e light target as described by Woodruff et al.[3] Diplopia recovery was defined at the time when the patients reported that they do not see double vision in any field any more. The recovery of ocular motility limitation was defined when ocular motility reaches full duction without any limitation, and graded as 0 by the ophthalmologist. The recovery of diplopia in the central 30 degrees was defined when diplopia disappeared within 30 degrees of the visual field confirmed by repeated tests.

Surgery was performed through transconjunctival or subciliary incision in case of orbital floor fracture and through transcaruncular approach or endoscopic endonasal approach in case of medial orbital wall fracture. Herniated or incarcerated orbital tissue was released and an implant was inserted to the fracture site. Forced duction test was performed at the end of the surgery to confirm whether tissue incarceration was released completely.

### Preoperative CT evaluation

Using CT imaging, fracture location, fracture type, fracture size, and presence/absence of tissue incarceration were evaluated. The orbital wall fracture size was calculated as reported by Lee et al.[14] Briefly, as for medial orbital wall fracture, the longest height on coronal image and longest length on axial image were measured, and as for orbital floor fracture, the longest horizontal length on coronal view and longest longitudinal length on sagittal view were measured. Orbital wall fractures were classified into two types; comminuted orbital wall fractures and linear non-displaced fractures (Trapdoor fractures). Comminuted fractures are characterized by the displacement of orbital bones and linear non-displaced fractures are defined by the lack of displacement of the involved bones. Fractures with tissue incarceration were divided according to CT findings whether the incarcerated tissue included the muscle belly or only the muscle sheath and/or fat tissue. Surgical records were also reviewed to confirm this categorization.

### Data analysis and statistics

For statistical analysis, SPSS ver. 21.0 software (IBM Corporation, Armonk, NY, USA) was used. The chi-square test was used to analyze categorical parameters between two groups, and the unpaired t test was used to analyze continuous parameters between two groups. Cumulative probabilities of recovery were assessed with the end point defined as 1) recovery of patient-reported diplopia 2) recovery of diplopia within central 30 degrees in all direction based on Goldmann diplopia field test,[15] and 3) complete recovery of ocular motility limitation. Factors affecting the recovery of diplopia and motility limitation were estimated using the log-rank test.

Univariate and multivariate cox proportional-hazard modeling were used to identify factors predictive of surgical outcome. The following factors were analyzed in univariate analysis: time interval from injury to first visit, time interval from first visit to surgery, time interval from injury to surgery, height and longest length of the medial orbital wall fracture, horizontal and longitudinal length of the orbital floor fracture, presence of trapdoor fracture, presence of muscle incarceration, presence of muscle sheath or fat incarceration, presence of retrobulbar hemorrhage and presence of fat or muscle herniation. Predictors with a P-value of 0.15 or less in univariate analysis were included as candidate variables in the multivariate analysis. A P-value < 0.05 was considered statistically significant. Data are presented as mean (standard deviation) unless stated otherwise.

## Result

### Patients characteristics

Among 244 pediatric patients diagnosed with orbital medial wall and/or floor fracture, 150 patients (61.5%) who underwent corrective surgery were included in this study. All 150 pediatric patients (136 boys [90.7%], 14 girls [9.3%]) were Korean and their age was 14.4 (±2.9; range, 5.7–17.9) years. Seventy one patients (47.3%) were diagnosed with orbital floor fracture, 45 (30.0%) with medial orbital wall fracture, and 34 (22.7%) with combined floor and medial wall fracture. The most common cause of injury was assault (50.7%) followed by bumping into something (18.0%), fall (12.7%), sports injury (10.7%), and motor vehicle accident (7.3%). Follow-up ranged from 2 weeks to 58.9 weeks (median, 9.8 weeks).

Mean time interval from trauma to first visit was 2.2 days (±5.8; range, 0.04–60.0 days) and time interval from first visit to surgery was 6.8 days (±15.6; range, 0.17–182.3). Mean time interval from trauma to surgery was significantly shorter in the trapdoor fracture group than non-trapdoor fracture group. (3.5 ± 4.1 days vs. 10.7 ± 19.1 days, P = 0.023). Symptom or signs at first visit were periorbital swelling (127 patients, 84.7%), ocular movement-induced pain (85 patients, 57.4%), nausea/vomiting (76 patients, 50.7%), ocular motility limitation (81 patients, 54.0%), and infraorbital hypesthesia (17 patients, 11.3%).

Among 45 patients with tissue incarceration, 38 (84.4%) presented with nausea/vomiting and 43 (95.6%) presented with eye movement-induced pain, showing that nausea/vomiting and eye movement-induced pain were very sensitive indicators of tissue incarceration.

Seventy-six patients (50.7%) reported binocular diplopia. Diplopia within the central 30 degrees was found in 49 (70%) of 70 patients who underwent the diplopia field test. Diplopia was most frequent in orbital floor trapdoor fracture (31/35, 89%) followed by medial wall trapdoor fracture (4/5, 80%), floor fracture (64/105, 61%) and medial orbital wall fracture (32/79, 41%).

Ocular motility was limited in 81 patients (54.0%) and supraduction limitation was most common (71 patients, 53%). Among 105 patients with floor fractures, 65 (61.9%) had supraduction limitation followed by infraduction limitation (24, 36.9%), abduction limitation (12, 11.4%) and adduction limitation (6, 5.7%). Among 79 patients with medial wall fractures, 26 (33%) had supraduction limitation followed by abduction limitation (8, 10%), adduction limitation (4, 5%), and infraduction limitation (4, 5%). Among 35 patients with orbital floor trapdoor fracture, 33 patients (94%) had supraduction limitation and 14 patients (42%) had infraduction limitation involving 2 patients without supraduction limitation. Among 5 patients with medial wall trapdoor fracture, 4 patients (80%) had abduction limitation and another 1 patient (20%) had supraduction limitation.

### CT findings

On CT-based evaluation, 38 patients (25.3%) had trapdoor-type fracture, 17 (11.5%) had muscle incarceration, and 29 (19.3%) had muscle sheath or fat incarceration. Retrobulbar hemorrhage was found in 11 patients (7.3%). In medial wall fractures, the mean longitudinal length was 5.5 mm (± 6.5; range, 0–23.5 mm) and mean height was 7.4 mm (± 8.8; range, 0–28.0 mm). In floor fractures, mean longitudinal length was 8.4 mm (± 8.6; range, 0–32.3 mm) and mean horizontal length was 6.4 mm (± 6.4; range, 0–23.3 mm).

### Factors associated with the presence of preoperative diplopia and ocular motility limitation

Patients were grouped according to the presence of diplopia and compared. The group with diplopia had shorter time interval between their first visit and surgery compared to the group without diplopia (P = 0.02, t-test). Nausea/vomiting, ocular movement-induced pain, ocular motility limitation, trapdoor fracture, muscle incarceration, fat or muscle sheath incarceration, and retrobulbar hemorrhage were more frequently founded in the group with diplopia. (All P < 0.05, chi-square test) (Table 1). Among patients without tissue incarceration, the group with diplopia had larger horizontal and longitudinal orbital floor fracture than the group without diplopia (P = 0.002 and 0.003, respectively).

**Table 1:** Comparison of preoperative characteristics between groups with and without diplopia.

Clinical characteristics	Preoperative diplopia (+)	Preoperative diplopia (-)	P value
Time interval from trauma to first visit (day)*	1.49 (2.41)	1.56 (2.10)	0.839
Time interval from trauma to surgery (day)*	5.86 (6.02)	8.17 (6.31)	**0.026**
Time interval from first visit to surgery (day)*	4.38 (5.27)	6.60 (6.17)	**0.020**
**Symptom and sign at first visit**			
Periorbital swelling	65 (85.5%)	61 (83.6%)	0.822
Infraorbital hypesthesia	6 (7.9%)	11 (15.3%)	0.200
Nausea/vomiting	57 (76%)	19 (26.4%)	**<0.001**
Ocular movement-induced pain	67 (90.5%)	17 (23.9%)	**<0.001**
Ocular motility limitation	70 (92.1%)	10 (13.7%)	**<0.001**
**CT findings**			
Trapdoor fracture^†^	35 (46.1%)	3 (4.2%)	**<0.001**
Muscle incarceration‡	17 (22.4%)	0 (0%)	**<0.001**
Fat or muscle sheath incarceration‡	24(32.0%)	4 (5.6%)	**<0.001**
Retrobulbar hemorrhage	11 (14.5%)	1 (1.4%)	**0.005**
**Patients without tissue incarceration**			
Height of medial wall fracture*^§^	5.80 (6.72)	7.52 (6.72)	0.200
Longitudinal length of medial wall fracture (mm)* ^§^	7.83 (8.76)	10.16 (9.44)	0.206
Horizontal length of floor fracture (mm)* ^§^	10.05 (7.20)	5.68 (6.39)	**0.002**
Longitudinal length of floor fracture (mm)* ^§^	13.39 (9.69)	7.50 (8.59)	**0.003**

CT = computed tomography. Factors with statistical significance are shown in boldface.

*Data are mean (standard deviation)

^†^Trapdoor fracture defined as the linear non-displaced fracture with the lack of displacement of the involved bones.

^‡^Fractures with tissue incarceration were divided according to CT findings whether the incarcerated tissue included the muscle belly or only the muscle sheath and/or fat tissue.

^§^Data included only patients without tissue incarceration.

Patients were grouped according to the presence of ocular motility limitation and compared. The group with ocular motility limitation had shorter time interval from their first visit to surgery (P = 0.024, t-test). Nausea/vomiting, ocular movement-induced pain, trapdoor fracture, muscle incarceration, fat or muscle sheath incarceration, and retrobulbar hemorrhage were more frequently founded in the group with ocular motility limitation than in the group without it. (All P < 0.05, chi-square test) (Table 2). Among patients without tissue incarceration, the group with ocular motility limitation had larger horizontal and longitudinal orbital floor fracture than the group without it (all P < 0.001).

**Table 2:** Comparison of preoperative characteristics between groups with and without ocular motility limitation.

Clinical characteristics	Motility limitation(+)	Motility limitation (-)	P value
Time interval from trauma to first visit (day)*	2.17 (6.89)	2.08 (4.22)	0.936
Time interval from trauma to surgery (day)*	6.35 (9.37)	12.72 (25.22)	**0.040**
Time interval from first visit to surgery (day)*	4.18 (4.99)	10.64 (24.73)	**0.024**
**Symptom and sign at first visit**			
Periorbital swelling	70 (86.4%)	45 (83.3%)	0.630
Infraorbital hypesthesia	10 (12.3%)	5 (9.3%)	0.781
Nausea/vomiting	60 (75%)	15 (27.8%)	**<0.001**
Ocular movement-induced pain	73 (91.3%)	11 (20.4%)	**<0.001**
**CT findings**			
Trapdoor fracture^†^	37 (45.7%)	1 (1.3%)	**<0.001**
Muscle incarceration‡	17 (21.0%)	0 (0%)	**<0.001**
Fat or muscle sheath incarceration‡	26 (32.5%)	2 (3.7%)	**<0.001**
Retrobulbar hemorrhage	11 (13.6%)	0 (0%)	**0.003**
**Patients without tissue incarceration**			
Height of medial wall fracture*^§^	5.36 (6.50)	7.87 (7.09)	0.079
Longitudinal length of medial wall fracture (mm)* ^§^	7.98 (9.19)	10.03 (9.48)	0.292
Horizontal length of floor fracture (mm)* ^§^	10.97 (6.95)	5.61 (6.29)	**<0.001**
Longitudinal length of floor fracture (mm)* ^§^	14.84 (9.17)	7.15 (8.36)	**<0.001**

CT = computed tomography. Factors with statistical significance are shown in boldface.

*Data are mean (standard deviation)

^†^Trapdoor fracture defined as the linear non-displaced fracture with the lack of displacement of the involved bones.

^‡^Fractures with tissue incarceration were divided according to CT findings whether the incarcerated tissue included the muscle belly or only the muscle sheath and/or fat tissue.

^§^Data included only patients without tissue incarceration.

### Factors associated with the recovery of diplopia

**Fig 1:**
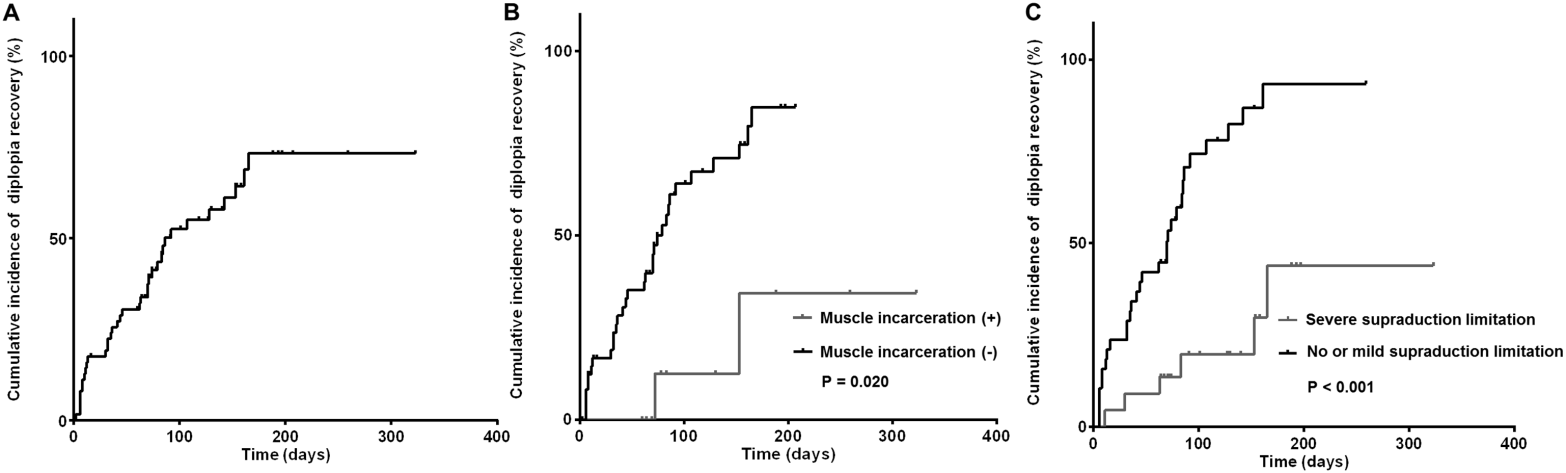
Kaplan-Meier curves of recovery of patient-reported diplopia. (A) Cumulative incidence of resolved patient-reported diplopia in pediatric patients with orbital wall fracture. The cumulative incidence of resolved diplopia at 3 months was 37.9% and diplopia was finally recovered in 34 patients (50.0%). (B) Univariate analyses on risk factors associated with patient-reported diplopia. Patients with muscle incarceration showed delayed recovery of diplopia compared to those without muscle incarceration. (C) Patients with severe supraduction limitation (grade -3 or -4) experienced delayed recovery of diplopia compared to those with no limitation or mild supraduction limitation (grade 0, -1 or -2).

Diplopia was resolved in 57% of patients at 323 days (median 86 days). In univariate analysis using long-rank test, patients who showed muscle incarceration and severe supraduction limitation (grade -3 or -4) showed late recovery of diplopia (P = 0.020, and < 0.001 respectively.) (Fig 1). In multivariate analysis using Cox proportional hazards model, the presence of severe supraduction limitation [hazard ratio (HR), 1.590; 95% CI, 1.217–2.078; P = 0.001], shorter time interval between trauma and first visit (HR, 0.733; 95% CI, 0.584–0.921; P = 0.008), and larger horizontal length of floor fracture (HR, 1.216; 95% CI, 1.074–1.376; P = 0.002) were independent risk factors of delayed diplopia recovery (Table 3).

**Table 3:** Factors associated with resolution of patient-reported diplopia.

	Univariate Analysis			Multivariate Analysis*		
	HR	95% CI	P value	HR	95% CI	P value
Time interval from trauma to first visit, *per 1day later*	0.850	0.685–1.055	0.141	0.733	0.584–0.921	**0.008**
Time interval from trauma to surgery, *per 1day later*	0.992	0.934–1.054	0.798			
Time interval from first visit to surgery, *per 1day later*	1.022	0.957–1.092	0.509			
Abduction limitation, per each grade	0.666	0.394–1.126	0.129			
Adduction limitation, per each grade	1.254	0.584–2.694	0.561			
Supraduction limitation, per each grade	1.590	1.217–2.078	**0.001**	1.743	1.193–2.546	**0.004**
Infraduction limitation, per each grade	1.030	0.760–1.396	0.847			
Trapdoor fracture^†^	5.308	1.266–22.252	**0.003**			
Muscle incarceration‡	1.969	0.970–3.997	0.061			
Fat or muscle sheath incarceration‡	0.775	0.401–1.499	0.449			
Retrobulbar hemorrhage	1.117	0.601–3.484	0.405			
Combined floor and medial wall fracture	1.277	0.607–2.683	0.519			
Height of medial wall fracture, *per 1mm larger*	0.973	0.913–1.037	0.400			
Longitudinal length of medial wall fracture, *per 1mm larger*	0.970	0.922–1.022	0.255			
Horizontal length of floor fracture, *per 1mm larger*	1.056	1.004–1.110	**0.035**	1.216	1.074–1.376	**0.002**
Longitudinal length of floor fracture, *per 1mm larger*	1.032	0.991–1.076	0.130			

CI = confidence interval; HR = Hazard ratio. Factors with statistical significance are shown in boldface.

* Variables with P < 0.15 in the univariate analysis were included in the multivariate model.

^†^Trapdoor fracture defined as the linear non-displaced fracture with the lack of displacement of the involved bones.

^‡^Fractures with tissue incarceration were divided according to CT findings whether the incarcerated tissue included the muscle belly or only the muscle sheath and/or fat tissue.

Regarding patients with central diplopia, central diplopia was resolved in 89.8% of patients at a median of 65 days. Multivariate analysis revealed that only the presence of supraduction limitation was an independent risk factor for delayed recovery of diplopia (HR, 1.549; 95% CI, 1.202–1.996; P = 0.001) (Table 4).

**Table 4:** Factors associated with resolution of central diplopia within 30 degree.

	Univariate Analysis			Multivariate Analysis*		
	HR	95% CI	P value	HR	95% CI	P value
Time interval from trauma to first visit, *per 1day later*	0.909	0.775–1.067	0.242			
Time interval from trauma to surgery, *per 1day later*	0.996	0.950–1.045	0.885			
Time interval from first visit to surgery, *per 1day later*	1.009	0.955–1.066	0.755			
Abduction limitation, per each grade	0.597	0.400–0.890	**0.011**			
Adduction limitation, per each grade	0.457	0.261–0.801	**0.006**			
Supraduction limitation, per each grade	1.596	1.245–2.047	**<0.001**	1.549	1.202–1.996	**0.001**
Infraduction limitation, per each grade	1.084	0.750–1.569	0.667			
Trapdoor fracture†	1.840	0.985–3.438	0.056			
Muscle incarceration‡	1.794	0.827–3.894	0.139			
Fat or muscle sheath incarceration‡	1.065	0.584–1.941	0.837			
Retrobulbar hemorrhage	1.443	0.633–3.288	0.383			
Combined floor and medial wall fracture	0.600	0.308–1.169	0.133			
Height of medial wall fracture, *per 1mm larger*	0.996	0.945–1.050	0.875			
Longitudinal length of medial wall fracture, *per 1mm larger*	0.992	0.995–1.029	0.653			
Horizontal length of floor fracture, *per 1mm larger*	1.034	0.987–1.083	0.161			
Longitudinal length of floor fracture, *per 1mm larger*	1.026	0.988–1.066	0.175			

CI = confidence interval; HR = Hazard ratio. Factors with statistical significance are shown in boldface.

* Variables with *P* < 0.15 in the univariate analysis were included in the multivariate model.

^†^ Trapdoor fracture defined as the linear non-displaced fracture with the lack of displacement of the involved bones.

^‡^Fractures with tissue incarceration were divided according to CT findings whether the incarcerated tissue included the muscle belly or only the muscle sheath and/or fat tissue.

### Factors associated with the recovery of ocular motility limitation

**Fig 2:**
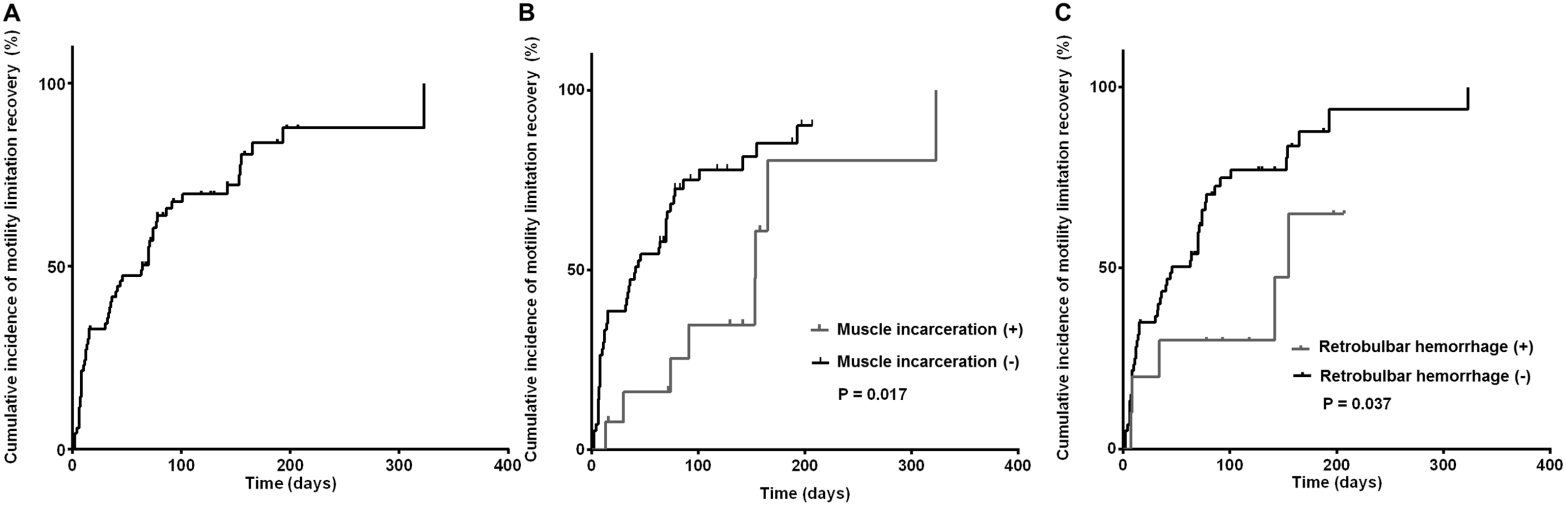
Kaplan-Meier curves of recovery of ocular motility limitation. (A) Cumulative incidence of resolved ocular motility in pediatric patients with orbital wall fracture. The cumulative incidence of resolved ocular motility at 3 months was 50.0%. In 58 patients (76.3%), ocular motility was finally recovered. (B) Univariate analyses on risk factors for delayed ocular motility limitation. Patients with muscle incarceration experienced more delayed recovery than those without muscle incarceration. (C) Patients with retrobulbar hemorrhage experienced more late recovery than those without retrobulbar hemorrhage.

Motility limitation was resolved in 74% of patients at 323 days (median 64 days). In the univariate analysis using long-rank test, patients with muscle incarceration and retrobulbar hemorrhage showed late recovery of ocular motility compared to those without muscle incarceration (P = 0.017, 0.037 respectively) (Fig 2). In multivariate analysis using Cox proportional hazards model, the presence of muscle incarceration (HR, 3.527; 95% CI, 1.542–8.071; P = 0.003), and retrobulbar hemorrhage (HR, 3.777; 95% CI, 1.453–9.819; P = 0.006) were independent risk factors for delayed recovery of ocular motility (Table 5).

**Table 5:** Factors associated with resolution of ocular motility limitation.

	Univariate Analysis			Multivariate Analysis*		
	HR	95% CI	P value	HR	95% CI	P value
Time interval from trauma to first visit, *per 1day later*	1.008	0.963–1.055	0.732			
Time interval from trauma to surgery, *per 1day later*	1.000	0.964–1.037	0.997			
Time interval from first visit to surgery, *per 1day later*	0.989	0.929–1.051	0.714			
Trapdoor fracture^†^	1.431	0.824–2.486	0.203			
Muscle incarceration‡	2.557	1.143–5.723	**0.022**	3.527	1.542–8.071	**0.003**
Fat or muscle sheath incarceration‡	0.752	0.432–1.306	0.316			
Retrobulbar hemorrhage	2.581	1.016–6.560	**0.046**	3.777	1.453–9.819	**0.006**
Combined floor and medial wall fracture	0.801	0.339–1.891	0.613			
Height of medial wall fracture, *per 1mm larger*	0.953	0.900–1.009	0.098			
Longitudinal length of medial wall fracture, *per 1mm larger*	0.972	0.934–1.010	0.148			
Horizontal length of floor fracture, *per 1mm larger*	1.026	0.983–1.071	0.293			
Longitudinal length of floor fracture, *per 1mm larger*	1.022	0.988–1.056	0.205			

CI = confidence interval; HR = Hazard ratio. Factors with statistical significance are shown in boldface.

* Variables with P < 0.15 in the univariate analysis were included in the multivariate model.

^†^ Trapdoor fracture defined as the linear non-displaced fracture with the lack of displacement of the involved bones.

^‡^Fractures with tissue incarceration were divided according to CT findings whether the incarcerated tissue included the muscle belly or only the muscle sheath and/or fat tissue.

## Discussion

This study presented the largest case series of 150 pediatric orbital wall fracture patients treated at one institution over 12 years. We revealed that in orbital wall fracture patients with diplopia, diplopia resolved later in patients with shorter time interval from trauma to first visit, severe preoperative supraduction limitation and larger horizontal length of floor fracture. In orbital wall fracture patients with ocular motility limitation, the limitation resolved later in patients with muscle incarceration and retrobulbar hemorrhage.

In patients with orbital wall fracture, diplopia or ocular motility limitation can be caused by extraocular muscle incarceration, direct muscle damage during the initial injury, or retrobulbar hemorrhage. In pediatric patients with orbital wall fracture, only a few studies have investigated factors associated with preoperative diplopia, ocular motility limitation and their postoperative recovery [16, 17]. Park et al.[16] reported that extended types of medial and orbital floor fractures were associated with preoperative diplopia. Su et al.[17] reported positive correlations between enophthalmos and preoperative diplopia, and between longer time interval from trauma to surgery and postoperative residual diplopia. However, these reports did not analyze the time to recovery of diplopia. To properly evaluate the prognostic factors for recovery of diplopia and ocular motility after surgery, we included the time points of recovery in the analysis.

In the current study, shorter time interval between trauma and first hospital visit was a negative prognostic factor for the recovery of diplopia. Early hospital visit may reflect severe symptoms including nausea/vomiting, which occurs by oculocardiac reflex from severe forms of orbital wall fracture such as muscle incarceration. Therefore, shorter time interval from trauma to the first visit actually reflects severe extraocular muscle injury, which causes delayed recovery of diplopia. A larger horizontal defect of orbital floor fracture was another poor prognostic factor for the recovery of diplopia. Larger orbital fractures more frequently accompany soft tissue herniation, resulting in stretch and translocation of the functional unit which causes motility limitation [18]. Yu et al.[8] demonstrated that the number of orbital wall fractures was inversely related to the recovery of diplopia; multiple fractures were associated with severe soft tissue damage resulting in delayed recovery [8]. However, since we excluded trapdoor fractures in our analysis of fracture size, it should not be interpreted that there is a linear relationship between recovery of diplopia and fracture size.

Regarding the recovery of diplopia in the central visual field, only severe supraduction limitation remained as a poor prognostic factor in multivariate analysis. As the central visual field within 30 degrees is important in daily living, supraduction limitation after pediatric orbital wall fracture can be considered as the most important factor affecting the patient's visual quality of life. In adult orbital wall fracture, most of the previous studies focused on the effectiveness of delayed surgery in the recovery of diplopia compared with surgery within 2 weeks, yet the prognostic value of ocular motility limitation in the recovery of diplopia had not been evaluated [19–21]. In addition, in pediatric patients without trapdoor fracture, there has been no previous analysis considering the effect of ocular motility limitation on the time to diplopia recovery.

Muscle incarceration and retrobulbar hemorrhage were poor prognostic factors for the recovery of motility limitation. Impaired blood supply to the extraocular muscle due to its incarceration can cause tissue necrosis and ischemic damage causing prolonged ocular motility limitation [22]. Iliff et al.[23] demonstrated that the longitudinal circulation of extraocular muscle was damaged irreversibly after a period of muscle incarceration. Nevertheless, surgical reduction of incarcerated muscle even in cases with severe motility limitation might restore the circulation and enhance recovery of motility limitation. Therefore, surgical release of incarcerated muscles can prevent permanent muscle damage and ocular motility limitation. Regarding retrobulbar hemorrhage, this study is the first to document the effect of retrobulbar hemorrhage on ocular motility limitation. We speculated that patients with retrobulbar hemorrhage tend to have more severe soft tissue damage and subsequent fibrosis, causing poorer motility outcomes. Indeed, in our study, children with retrobulbar hemorrhage had a higher incidence of multiple orbital fractures including superior or lateral orbital walls, probably causing severe soft tissue damage.

Trapdoor type fracture was the most common type of orbital wall fracture in patients with diplopia and motility limitation. Trapdoor fracture was well known to be more common in the pediatric population and especially in younger children [10, 24]. The proportion of trapdoor fracture in our study was 25.3% which is lower than that of previous reports (27.8–93%) because of the higher mean age in our study [2, 7, 25, 26].

Numerous authors have advocated that earlier surgical intervention for children with muscle incarceration results in better outcomes than later intervention [26–30]. Gerbino et al.[29] reported that patients who underwent surgery within 24 hours showed a lower incidence of postoperative residual diplopia than patients who underwent surgery after 24 hours. Jordan et al.[27] recommended surgery within a few days of injury for fractures with ocular motility limitation. However, in our current study, no correlation was found between the recovery of diplopia or ocular motility limitation and the time of surgery. The suggested reason is that most of our surgeries were performed within 3 days of hospital visit in cases of trapdoor fracture, in contrast to the previous reports which had performed surgery as long as 12 days after injury, even in cases with trapdoor fracture [26],[28]. However, the theoretical concerns associated with prolonged impairment of blood flow should not be overlooked only by these results.

Limitations of this study include the retrospective nature of the review, subjective methods of diplopia evaluation, and incomplete pre- and post-operative documentation of visual fields with diplopia. In addition, this study has a lack of uniform follow-up after presentation. Finally, we limited inclusion criteria for patients with medial and/or inferior orbital wall fracture. This limits the analysis, as many pediatric orbital fractures involve the superior wall [31]. However, since the orbital roof fractures are the least likely to require intervention, it would have little effect on our study, which only analyzed patients who underwent surgery.

In conclusion, pediatric orbital wall fractures with supraduction limitation, muscle incarceration, large orbital floor fracture, or retrobulbar hemorrhage tend to show late recovery of diplopia and ocular motility limitation. Recovery of diplopia and ocular motility limitation could be significantly delayed even after successful surgery because it is associated with damage on soft tissue and extraocular muscle combined with bony fracture. Therefore careful ophthalmic examination and CT evaluation is important to predict surgical outcomes in pediatric patients with orbital wall fracture.
